# Supplementary material for: Subcellular Compartmentalization of Glucose Mediated Insulin Secretion
Source: Cells. 2025 Jan 29;14(3):198. doi: 10.3390/cells14030198 (PMC11817236; doi:10.3390/cells14030198)
Supplement: Supplementary file 1 [file cells-14-00198-s001.zip › cells-3380020-supplementary.pdf]

SUPPLEMENTAL INFORMATION

Article

Subcellular Compartmentalization of Glucose Mediated Insulin Secretion

Zhongying Wang [1,2], Tatyana Gurlo [3], Leslie S. Satin [4], Scott E. Fraser [1,2] and Peter C. Butler [3,*]

[1] Translational Imaging Center, Michelson Center for Convergent Bioscience, University of Southern California, Los Angeles, CA 90089, USA; zhongyingw7@gmail.com (Z.W.); sfraser@provost.usc.edu (S.E.F.)

[2] Dana and David Dornsife College of Letters, Arts and Sciences, University of Southern California, Los Angeles, CA 90089, USA

[3] Larry L. Hillblom Islet Research Center, David Geffen School of Medicine at UCLA, Los Angeles, CA 90089, USA; tgurlo@g.ucla.edu

[4] Brehm Diabetes Center, Caswell Diabetes Institute, Department of Pharmacology, University of Michigan, Ann Arbor, MI 38105, USA; lsatin@umich.edu

* Correspondence: pbutler@mednet.ucla.edu

Supplemental Methods

Organelle Live Staining

Mitochondria were labeled by MITO-ID® Red dye at 10,000 dilutions in TCM for 30 min, at 37°C, 5% CO_2, for mitochondria net migration test.

Mitochondria net migration test

The fluorescence intensity data were thresholded and masked to determine the cell area and mitochondria area within a single section. A region of interest (ROI) was selected as a rectangular area along the longest axis of the cell with a width of 2 μm. This rectangular area was equally divided into five sections along the long axis. The regions closest to the submembrane and perinuclear areas, comprising 20% of the ends of the rectangle, were selected as ROIs. The ratio of mitochondria area to cell area was analyzed within these ROIs and represented as fractions.

Supplemental Figures

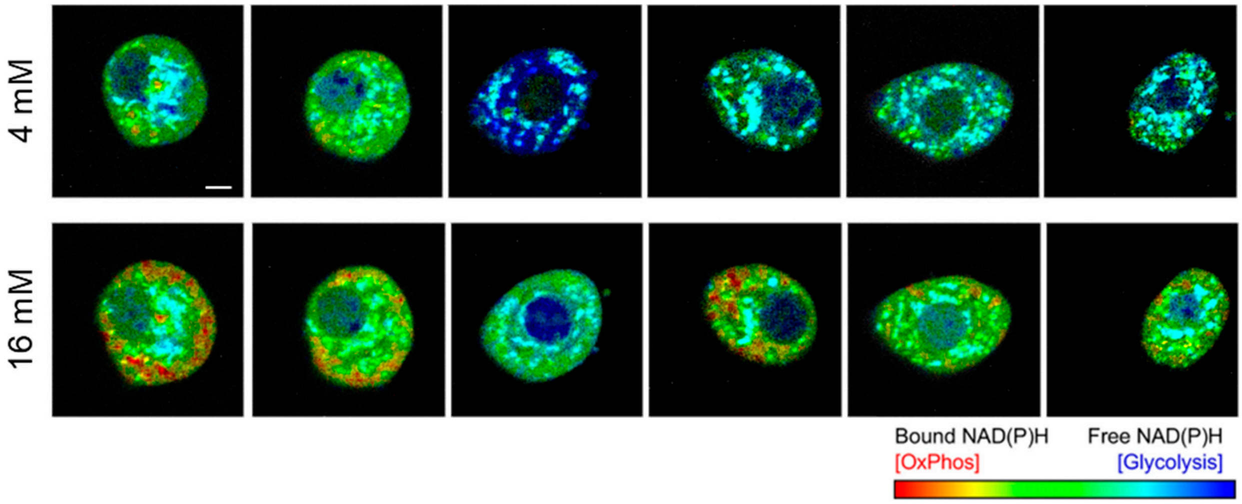

Figure S1. Representative FLIM images of mouse primary beta cells illustrate OxPhos initially enhanced at peripheral area of the cell (5 min after stimulation with 16 mM glucose). Scale bar 5 μm applies to all images.

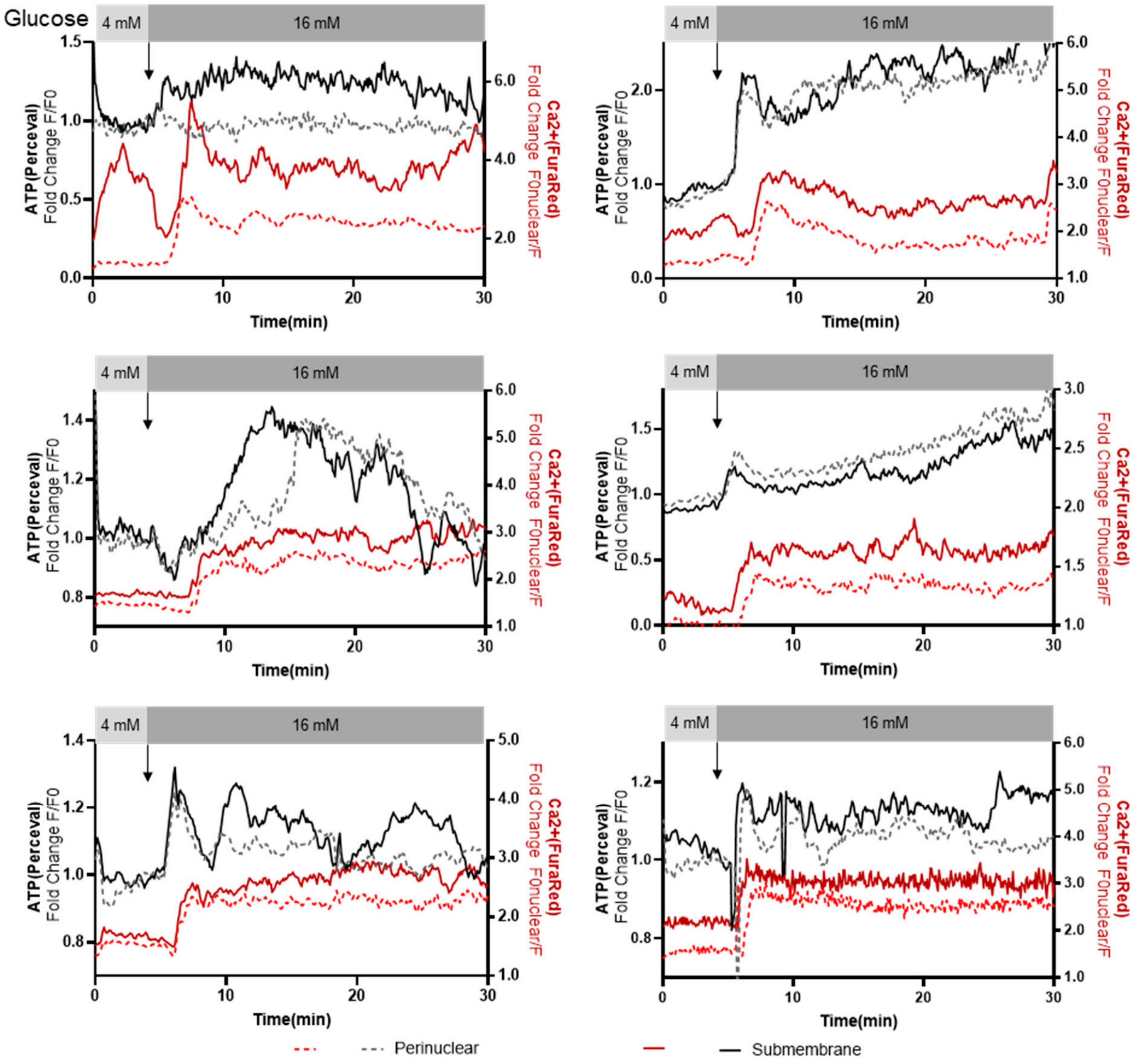

Figure S2. Representative ATP (black) and calcium (red) response of six primary mouse beta cells to glucose stimulation (16 mM) in perinuclear region (dashed line) and submembrane region (solid line). Arrows indicate the addition of high glucose.

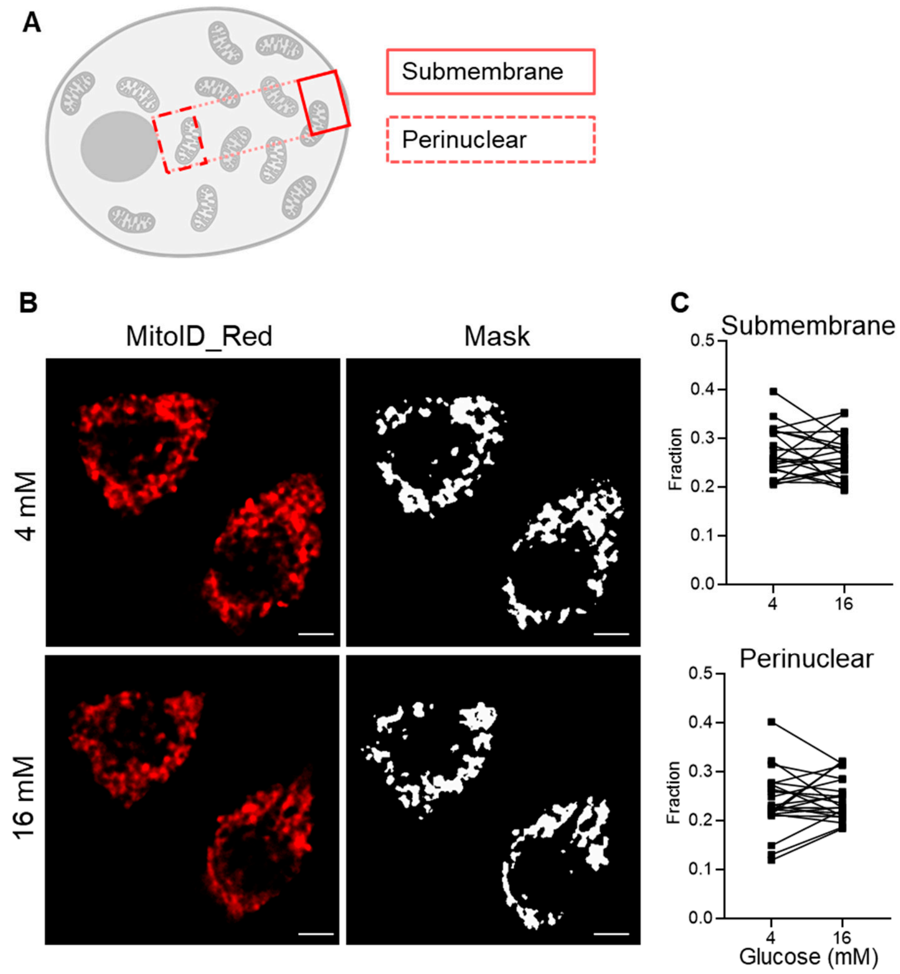

Figure S3. No net migration of mitochondria to the submembrane region upon glucose exposure. A, diagram of mitochondria population analysis over the long polar axis. B, representative images of INS cell line stained by MitoID _Red dye to track the morphology of mitochondria under low glucose and after high glucose stimulation (Left). The mask of mitochondria staining was applied to measure mitochondria area (Right). C, there was no change of mitochondria area at periphery of the cell or at peri-nucleus region upon stimulation with glucose. All data points connected by straight lines represent paired measurements made on the same cell or position. Wilcoxon signed-rank test was used. Significance was assumed at $p < 0.05$.
